# Autophagy and Apoptosis in Hepatocellular Carcinoma Induced by EF25-(GSH)_2_: A Novel Curcumin Analog

**DOI:** 10.1371/journal.pone.0107876

**Published:** 2014-09-30

**Authors:** Tao Zhou, Lili Ye, Yu Bai, Aiming Sun, Bryan Cox, Dahai Liu, Yong Li, Dennis Liotta, James P. Snyder, Haian Fu, Bei Huang

**Affiliations:** 1 School of life Sciences, Anhui University, Hefei, China; 2 Department of Chemistry, Emory University, Atlanta, Georgia, United States of America; 3 Emory Institute for Drug Development (EIDD), Emory University, Atlanta, Georgia, United States of America; 4 Department of Pharmacology and Emory Chemical Biology Discovery Center, Emory University, Atlanta, Georgia, United States of America; 5 Center for Stem Cell and Translational Medicine, Anhui University, Hefei, China; Taipei Medicine University, Taiwan

## Abstract

Curcumin, a spice component as well as a traditional Asian medicine, has been reported to inhibit proliferation of a variety of cancer cells but is limited in application due to its low potency and bioavailability. Here, we have assessed the therapeutic effects of a novel and water soluble curcumin analog, 3,5-bis(2-hydroxybenzylidene)tetrahydro-4*H*-pyran-4-one glutathione conjugate [EF25-(GSH)_2_], on hepatoma cells. Using the MTT and colony formation assays, we determined that EF25-(GSH)_2_ drastically inhibits the proliferation of hepatoma cell line HepG2 with minimal cytotoxicity for the immortalized human hepatic cell line HL-7702. Significantly, EF25-(GSH)_2_ suppressed growth of HepG2 xenografts in mice with no observed toxicity to the animals. Mechanistic investigation revealed that EF25-(GSH)_2_ induces autophagy by means of a biphasic mechanism. Low concentrations (<5 µmol/L) induced autophagy with reversible and moderate cytoplasmic vacuolization, while high concentrations (>10 µmol/L) triggered an arrested autophagy process with irreversible and extensive cytoplasmic vacuolization. Prolonged treatment with EF25-(GSH)_2_ induced cell death through both an apoptosis-dependent and a non-apoptotic mechanism. Chloroquine, a late stage inhibitor of autophagy which promoted cytoplasmic vacuolization, led to significantly enhanced apoptosis and cytotoxicity when combined with EF25-(GSH)_2_. Taken together, these data imply a fail-safe mechanism regulated by autophagy in the action of EF25-(GSH)_2_, suggesting the therapeutic potential of the novel curcumin analog against hepatocellular carcinoma (HCC), while offering a novel and effective combination strategy with chloroquine for the treatment of patients with HCC.

## Introduction

Hepatocellular carcinoma (HCC) is the fifth most common cancer and the third leading cause of cancer death worldwide [1]. Certain regions in Asia and Africa are disproportionally affected, while China alone accounted for half of the new liver cancer cases occurring worldwide during 2008 [2]. Chemotherapy plays a crucial role in the treatment of HCC especially at advanced stages when curative therapies like resection and liver transplantation are inapplicable [3], [4]. However, since most widely used chemotherapeutic drugs show severe side effects, development of novel and safe agents is mandatory.

Curcumin, a natural compound isolated from the commonly used spice turmeric, has been shown to inhibit cell proliferation in various types of cancer cells *in vitro* and *in vivo* [5]. Numerous curcumin derivatives and analogues have been developed in recent years in order to enhance anti-tumor efficacy and overcome limitations such as poor aqueous solubility, relative low bioavailability and intense yellow staining [6], [7]. The compounds 3,5-bis(2-flurobenzylidene)piperidin-4-one (EF24) and 3,5-bis(pyridin-2-ylmethylene)piperidin-4-one (EF31), synthetic structural analogues of curcumin [8], exhibit improved anticancer activity and a safety profile similar to curcumin [8]–[11]. Synthetic manipulation of these agents generates EF24-(GSH)_2_ and EF31-(GSH)_2_, double glutathione conjugates with no less anticancer capability compared to EF24 and EF31. However the conjugates exhibit superior stability in solution, water solubility and lack of color [8]. The structurally related compound 3,5-bis(2-hydroxybenzylidene)tetrahydro-4*H*-pyran-4-one (EF25) and its double glutathione conjugate EF25-(GSH)_2_, are under investigation and reported here for the first time.

Although much of the research into the anti-cancer mechanisms of curcumin has focused on its ability to induce apoptosis, curcumin has also been found to induce other types of cell death including autophagic cell death, mitosis catastrophe and paraptosis [5], [12]–[14]. By testing different cell lines, it has been found that the mode of cell death induced by curcumin varies among different cell lines, and the mechanisms of different cellular responses remains a mystery [14].

Here we show that *in vitro* EF25-(GSH)_2_ exhibits preferential toxicity to malignant liver cancer cells compared with immortalized human hepatic cells. In parallel, *in vivo* EF25-(GSH)_2_ significantly suppresses the growth of hepatocellular carcinoma (HepG2) xenografts and is relatively nontoxic to mice. Further investigation into the mechanism of action reveals that EF25-(GSH)_2_ induces a mixed mode of cell death in hepatoma cells in which autophagy, cell cycle arrest, cytoplasmic vacuolization, caspase-dependent and caspase-independent apoptosis all take place.

## Materials and Methods

### 1. Ethics Statement

All procedures involving mice were approved by Anhui Medical University Animal Care Committee, which follows the protocol outlined in The Guide for the Care and Use of Laboratory Animals published by the USA National Institute of Health (NIH publication No. 85-23, revised 1996). The details of animal welfare and steps taken to ameliorate suffering were in accordance with the recommendations in The Guide for the Care and Use of Laboratory Animals, and all efforts were made to minimize suffering.

### 2. Reagents

Cisplatin was purchased from the National Institutes for Food and Drug Control (China). Curcumin and other reagents were purchased from Sigma-Aldrich. Antibodies against microtubule-associated protein 1 light chain 3B (LC3B), caspase-3, caspase-8 and actin were obtained from Cell Signaling Technology. mCherry-GFP-LC3B plasmid was kindly provided by Dr. Mian Wu (University of Science and Technology of China). Lentivirus-based shRNA constructs targeting the human Atg5 gene (pLKO.1-shAtg5-D8 and pLKO.1-shAtg5-D9, targeting different sequences), human Beclin-1 gene (pLKO.1-shBeclin-1-C2 and pLKO.1-shBeclin-1-C3, targeting different sequences) were kindly provided by Dr. Qinghua Shi (University of Science and Technology of China), and negative control targeting LacZ (pLKO.1-shLacZ) was obtained from the National RNAi Core Facility (Taiwan). Three helper plasmids (pLP1, pLP2 and pLP/VSVG) of lentiviral systems were kindly provided by Dr. Yong Li (Anhui University).

### 3. Synthesis of EF25 and EF25-(GSH)_2_

EF25 was prepared as previously reported where it was originally named “compound 11” [15], while EF25-(GSH)_2_ was obtained by a procedure identical to that for EF24-(GSH)_2_ [8]. It should be noted that EF25 combined with glutathione much more slowly by comparison with EF24. EF25 (64.0 mg, 0.2 mmol, 1.0 eq.) in CH_3_CN (0.2 ml) was added dropwise to a solution of GSH (123.0 mg, 0.4 mmol, 2.0 eq.) in water at room temperature. The reaction mixture was refluxed for 2 hr until the disappearance of both the yellow color and EF25 by LC/MS. Evaporation of the solvent delivered the product as a white powder in quantitative yield. HR-ESI-MS (*m/z*): [M+H]^+^ calcd for C_39_H_51_O_16_N_6_S_2_ 923.28053, found, 923.28121 ( = 00068 amu) (Fig. S1).

The ^1^H NMR spectrum of EF25-(GSH)_2_ in DMSO-*d6* and D_2_O (buffer pH7) are complex due to the presence of diastereoisomers resulting from GSH conjugation at the two C = C bonds of EF25. The ^1^H NMR spectrum of the unconjugated EF25 in DMSO-*d6* exhibits a sharp singlet at 7.89 ppm assigned to the olefinic(C = )C–H proton and sharp aromatic signals at 6.8–7.3 ppm. The intensity of the olefinic signal decreases for the conjugated EF25-(GSH)_x_, and the sharp aromatic signals observed for unconjugated EF25 are broadened for EF25-(GSH)_x_. These observations indicate a mixture of the mono- and bis-conjugates EF25-(GSH) and EF25-(GSH)_2_, respectively, and possibly rapid exchange between them (Fig. S2). The comparison of the ^1^H NMR spectra of EF25 in DMSO-*d6* and EF25-(GSH)_2_ in D_2_O (pH7) illustrates the absence of observable quantities of unconjugated EF25 (Fig. S3). Thus, in these solvents, the equilibrium lies primarily on the side of the conjugates, although in biological tissues it is shifted to the unconjugated form as the hydrophobic EF25 interacts with its target proteins.

### 4. Cell culture

The three human hepatocellular carcinoma cell lines (HepG2, SMMC-7721 and BEL-7402) and one immortalized human hepatic cell line (HL-7702) were kindly supplied by Dr. Hui Zhong (Academy of Military Medical Sciences) [16]–[19]. The other three human tumor cell lines (HCT116 human colon cancer cell line, A549 human lung carcinoma cell line and Hela human cervical carcinoma cell line) and HEK293FT cell line were kindly supplied by Dr. Qinghua Shi (University of Science and Technology of China) [20], [21]. The HepG2, HCT116, A549, Hela, BEL-7402 and HEK293FT cells were grown in DMEM (Gibco). The SMMC-7721 cells and HL-7702 cells were grown in RPMI 1640 (Gibco). Both media were supplemented with 10% fetal bovine serum (FBS; Gibco), 100 units/mL penicillin and 100 µg/mL streptomycin at 37°C in a humidified incubator containing 5% CO_2_.

### 5. Cell viability assay

Cells (8×10^3^ per well) were seeded onto 96-well plates in supplemented DMEM and incubated overnight. Then the cells were treated in triplicate for the indicated time with increasing doses of EF25-(GSH)_2_ in 10% FBS containing DMEM or RPMI 1640 without antibiotic. Treated cells were then incubated in the presence of 0.5 mg/mL 3-(4,5-Dimethylthiazol-2-yl)-2,5-diphenyltetrazoliumbromide (MTT) for 4 h. The formazan crystals were dissolved in DMSO and monitored at an absorbance of 490 nm. Absorbance values were normalized to those obtained for the untreated cells to determine percentage survival. All experiments were repeated at least three times. IC_50_ values (50% inhibition concentration) were then calculated using the Statistical Package for the Social Sciences (SPSS, Inc.).

### 6. Colony formation assay

Twenty-four-well plates were seeded with 500 viable cells in complete medium and incubated overnight. The cells were then treated in triplicate with EF25-(GSH)_2_ in 10% FBS containing DMEM without antibiotic for 24 h. The compound-containing medium was then removed, and the cells were washed with PBS twice and incubated in complete medium for another two weeks. Medium was replaced once at the end of the first week. The cell colonies formed were fixed in 10% formalin for 10 min and visualized by staining with Giemsa [22].

### 7. DNA content analysis

5×10^6^ HepG2 cells were seeded into six-well plates and incubated overnight. The cells were treated with EF25-(GSH)_2_ and then collected by trypsinization and fixed in precooled 70% ethanol overnight. Cells were then stained with 50 µg/mL propidium iodide (PI) in the presence of 100 µg/mL RNase A. DNA content was analyzed by FACSCalibur (Becton Dickinson), and data were analyzed by the Flowjo software. The percentage of cells in sub-G_1_-G_0_ was used to represent the apoptosis rate.

### 8. HepG2 cell tumor xenograft in mice

Five-week-old male athymic mice were obtained from Beijing Vital River Laboratory Animal Co., Ltd. Animals were given ad libitum access to water and standard mouse chow. 5×10^6^ HepG2 cells were injected subcutaneous into the left flank and allowed to form a xenograft. Treatment was initiated when the tumor reached a group mean of 100 mm^3^. EF25-(GSH)_2_ was dissolved in PBS and administrated i.p. daily at a dose of 1.5 mg/kg body weight for 30 days [23], [24]. The control group was given the same volume of PBS only. Tumor volume was calculated using the formula V = a^2^×b/2, where a and b represent the shorter and longer diameters of the tumor, respectively. At the end of the treatment, the mice were sacrificed under etherization, and the tumors were weighed.

### 9. Transmission electron microscopy

Treated cells were collected by trypsinization, washed twice with PBS, and then fixed with ice-cold 3% glutaraldehyde in 0.1 mol/L cacodylate buffer at 4°C overnight. Cells were then postfixed in osmium tetroxide and embedded in Polybed resin. Ultrathin sections were double stained with uranyl acetate and lead citrate and examined with a JEM-2100 electron microscope.

### 10. 4, 6-diamidino-2-phenylindole (DAPI) staining

At the end of the EF25-(GSH)_2_ treatment, DAPI was added to the medium at a final concentration of 1 µg/mL. After staining with DAPI for 15 min, cell morphology was examined by laser confocal microscopy. Uniformly stained nuclei with clear margins were regarded as normal, while condensed or fragmented nuclei with strengthened fluorescence were considered apoptotic.

### 11. Transient transfection

HepG2 cells were seeded in six-well plates and incubated overnight. mCherry-GFP -LC3 was transfected using Lipofectamine 2000 according to the manufacturer's instructions. The transfection mixture was replaced with 10% FBS containing DMEM without antibiotic 6 hours after transfection and incubated for another 24 hours. Cells were then treated with EF25-(GSH)_2_ for the indicated time. Green (GFP) and red (mCherry) fluorescence was observed under a laser confocal microscope.

### 12. Knockdown of Atg5 and Beclin-1 expression by lentivirus-delivered shRNA

For lentivirus preparation, HEK293FT cells were transfected with pLKO.1-shRNA and three helper plasmids (pLP1, pLP2 and pLP/VSVG) with Fugene 6 Reagent (Roche). To generate human Atg5-knockdown or Beclin-1-knockdown cells, HepG2 cells were transduced with lentivirus expressing shAtg5 or shBeclin-1, respectively, and selected with 2 µg/ml puromycin.

### 13. Western blot assay

Cell lysates were subjected to SDS-PAGE and blotted onto polyvinylidene difluoride (PVDF) membranes. The membranes were then incubated with the each primary antibody and appropriate secondary antibody. The immunoblots were visualized by a chemiluminescence HRP substrate.

### 14. Statistical analysis

All values are expressed as the mean±SE. Data were analyzed using two-tailed student's t test. P≤0.05 was considered statistically significant. Statistical analysis was performed using SPSS.

## Results

### 1. EF25-(GSH)_2_ inhibited proliferation of tumor cells

**Figure 1:**
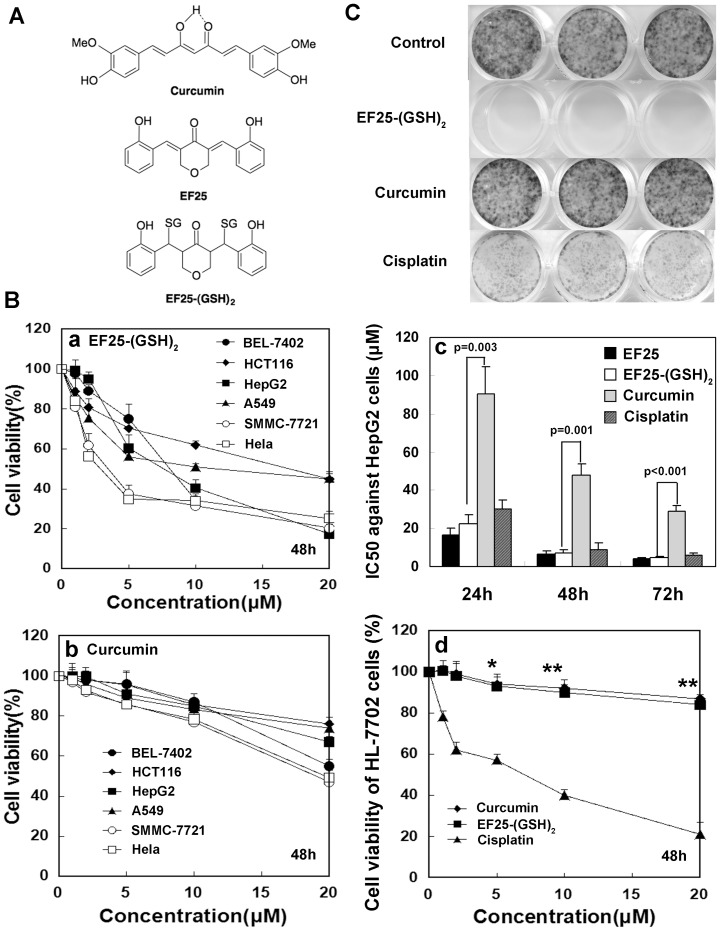
EF25-(GSH)2 inhibited proliferation of tumor cells *in vitro*. (A) The structures of curcumin, EF25 and EF25-(GSH)_2_. (B) *a and b*, EF25-(GSH)_2_ showed similar toxicity towards six human tumor cells (BEL-7402, HCT116, HepG2, A549, SMMC-7721 and Hela) (*a*) and the toxicity of curcumin was much lower than that of EF25-(GSH)_2_ (*b*). *c*, cells were incubated with increasing doses of indicated compounds for 24-, 48-, and 72-h periods and analyzed by MTT assay. The IC_50_ of each agent at each time period was calculated and compared using SPSS. The IC_50_ of EF25-(GSH)_2_ is much lower than that of curcumin and essentially equivalent to that of cisplatin. *d*, the cytotoxicity of EF25-(GSH)_2_ to HL-7702 cells was much lower than that of cisplatin and similar to curcumin after 48-hour incubation as determined by MTT assay (*, p<0.01, **, p<0.001). (C) Cells were incubated with 0.5 µmol/L of the indicated compound for 24 h and subsequently allowed to grow into colonies (2 weeks). EF25-(GSH)_2_ totally inhibited colony formation leading to clean plates, while curcumin and cisplatin did not. Results are representative of three independent experiments.

The structures of curcumin analogues, EF25 and EF25-(GSH)_2_, examined in this study are presented in Figure 1A. We first determined the effect of EF25, EF25-(GSH)_2_ and curcumin on cell proliferation of HepG2 cells. Cisplatin, a widely used chemotherapeutic drug, was also examined under the same conditions. EF25-(GSH)_2_, which is far more effective than curcumin, showed similar cytotoxicity to cell lines derived from three types of hepatomas (HepG2, SMMC-7721 and BEL-7402) and three other carcinomas (HCT116, A549 and Hela) (Fig. 1B a, b). EF25 and EF25-(GSH)_2_ exhibit similar cytotoxicity in a dose- and time-dependent manner, indicating that GSH association does not change the cell-kill capacity of the EF25 conjugate. The mechanism of this phenomenon was elucidated in our previous investigation showing that the conjugate is reversible [8]. Thus, the active agent in both cases would appear to be EF25. That the latter showed slightly better activity than EF25-(GSH)_2_ at 24 h, and that the difference between them diminished as time prolonged to 72 h (Fig. 1B c), is mostly likely due to differential cell penetration regulated in part by the equilibrium shift from conjugate to free EF25. In effect, EF25-(GSH)_2_ serves as a pro-drug capable of releasing the active agent EF25 by reversal of the well-known Michael reaction in cells [8]. The cytotxicity of EF25-(GSH)_2_ to HepG2 cells is much greater than that of curcumin as characterized by its much lower IC_50_ value (7.2 µmol/L at 48 h), which is close to that of cisplatin (9.1 µmol/L at 48 h) (Fig. 1B c). In order to examine if EF25-(GSH)_2_ can preferentially kill malignant cells, cytotoxicities of the conjugate and cisplatin against immortalized human hepatic cell line HL-7702 were examined at 48 h post-treatment by the MTT assay. The results show that the cytotoxicity of EF25-(GSH)_2_ to HL-7702 cells was much lower than that of cisplatin (Fig. 1B d).

To examine the long-term effect of EF25-(GSH)_2_ treatment, the colony formation assay was performed. A quantity of 0.5 µmol/L EF25-(GSH)_2_ totally inhibited colony formation, while 0.5 µmol/L curcumin had nearly no effect. An 0.5 µmol/L cisplatin treatment also caused a significant reduction in colony number, but was much less efficiently by comparison with EF25-(GSH)_2_ (Fig. 1C).

### 2. EF25-(GSH)_2_ suppresses HepG2 xenograft growth

**Figure 2:**
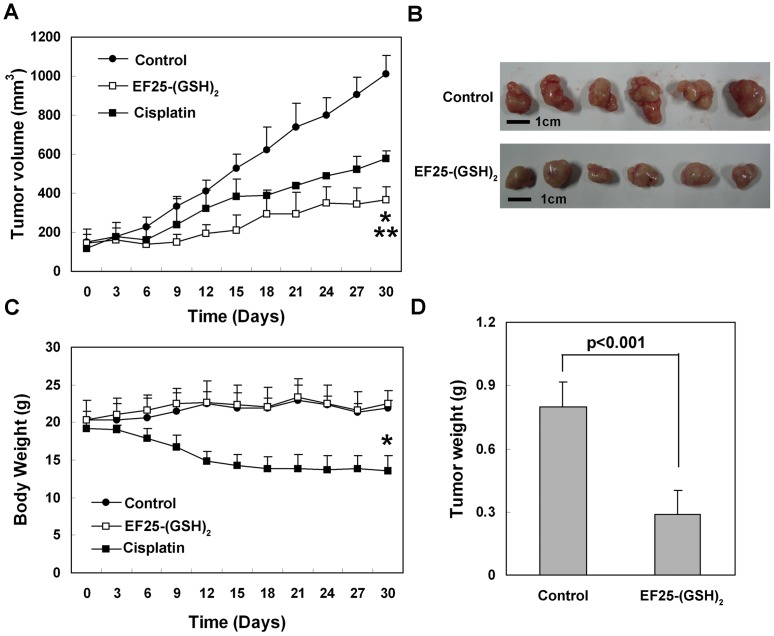
EF25-(GSH)_2_ suppressed HepG2 xenograft growth *in vivo*. (A) HepG2 cells were injected into the left flank of nude mice and tumors were allowed to grow to a size of about 100 mm^3^. Subsequently, EF25-(GSH)_2_ (dissolved in PBS, 1.5 mg/kg body weight) was injected daily i.p. for 30 d (n = 6). The cisplatin group (dissolved in PBS, 0.5 mg/kg body weight) was injected every other day i.p. (n = 4), and the control group was injected with the same volume of PBS daily i.p. (n = 6). Tumor growth was significantly suppressed in the EF25-(GSH)_2_-treated group compared to either control (**, p<0.001) or cisplatin-treated group (*, p<0.01). (B) At the end of the treatment, tumor volume in the EF25-(GSH)_2_-treated group was much smaller than that of the control group. (C) The EF25-(GSH)_2_-treated group maintained normal weight gain while the cisplatin-treated group suffered a remarkable weight loss throughout the treatment (*, p<0.001). (D) At the end of the treatment, EF25-(GSH)_2_ treatment resulted in significantly lower tumor weight when compared with control group.

To assess the antitumor potential of EF25-(GSH)_2_ *in vivo*, HepG2 xenograft-bearing mice were given EF25-(GSH)_2_ i.p. daily for 30 days (1.5 mg/kg body weight, PBS as vehicle, n = 6). For comparison, cisplatin was dissolved in PBS and given i.p. every other day at a dose of 0.5 mg/kg body weight (n = 4). Compared with vehicle treatment (n = 6), EF25-(GSH)_2_ treatment significantly suppressed the growth of tumor volume which was much more efficient than cisplatin treatment (Fig. 2A, B). Notably, while the EF25-(GSH)_2_-treated group maintained normal weight gain, the cisplatin-treated animals suffered a remarkable weight loss throughout the treatment (Fig. 2C). The tumor weights of EF25-(GSH)_2_-treated mice were also significantly lower than that of the control group (Fig. 2D). There was no apparent change in liver, kidney and spleen weight in the EF25-(GSH)_2_-treated group, while the weight of these organs dropped dramatically in the cisplatin-treated group (data not shown).

### 3. The morphological appearance of EF25-(GSH)_2_-treated HepG2 cells

**Figure 3:**
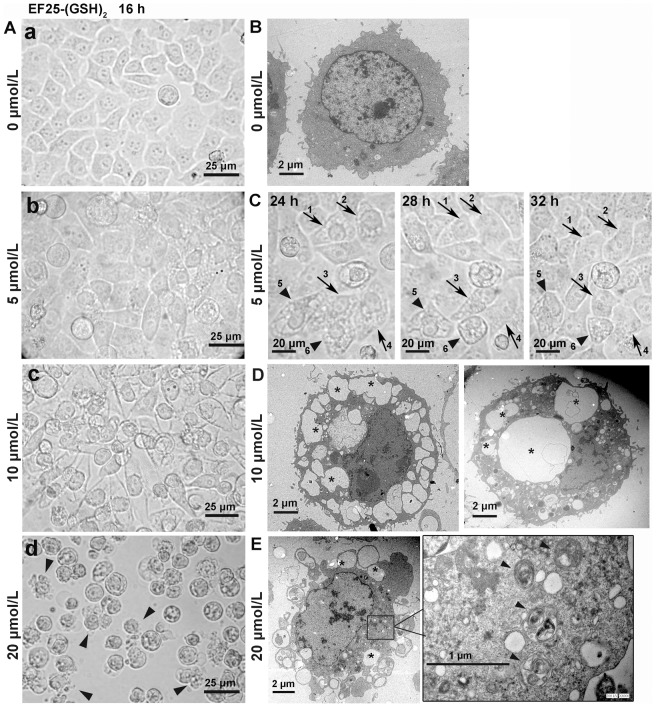
The morphological appearance of EF25-(GSH)_2_-treated HepG2 cells. (A) HepG2 cells treated with increasing concentrations of EF25-(GSH)_2_ for 16 h were observed under a light microscope and representative images were visualized. EF25-(GSH)_2_-treated cells underwent vacuolization, the extent of which varied when treated with different concentrations of EF25-(GSH)_2_. At 20 µmol/L, apoptotic-like cell membrane blebbing was observed (arrowheads). (B) A representative transmission electron microscopy (TEM) image of untreated HepG2 cells. (C) In 5 µmol/L EF25-(GSH)_2_-treated cells, most vacuolated cells regained normal morphology at 32 h post-treatment (arrows, 1-4) while some did not (arrow heads, 5 and 6). (D) Representative TEM images of cells treated with 10 µmol/L EF25-(GSH)_2_ for 16 h. *, large empty vacuoles with varying size. (E) Representative TEM images of cells treated with 20 µmol/L EF25-(GSH)_2_ for 16 h. *, large empty vacuoles; arrows, autophagic vacuoles.

The morphological changes in EF25-(GSH)_2_-treated cells were observed under a light microscope to observe apparent vacuolization in the cytoplasm. The number of cells that suffered cytoplasmic vacuolization and its extent varied when treated with different concentrations of EF25-(GSH)_2_, but all reached a maximum at about 16 hours post-treatment (Fig. 3A). When treated with 5 µmol/L EF25-(GSH)_2_, the cells experienced moderate vacuolization which regained normal morphology after about 8 hours (Fig. 3C). In contrast, most cells exposed to 10 µmol/L EF25-(GSH)_2_ showed extensive and irreversible vacuolization in the cytoplasm. At 20 µmol/L, EF25-(GSH)_2_ not only induced massive cytoplasmic vacuolization but also caused apoptotic membrane blebbing (Fig. 3A).

In the cytoplasm of HepG2 cells treated with 10 or 20 µmol/L EF25-(GSH)_2_ for 16 hours, large vacuoles of varying size were content-free and single membrane bounded, while the small vacuoles resemble autophagic vacuoles (Fig. 3D, E).

### 4. EF25-(GSH)_2_ induced autophagy in HepG2 cells

**Figure 4:**
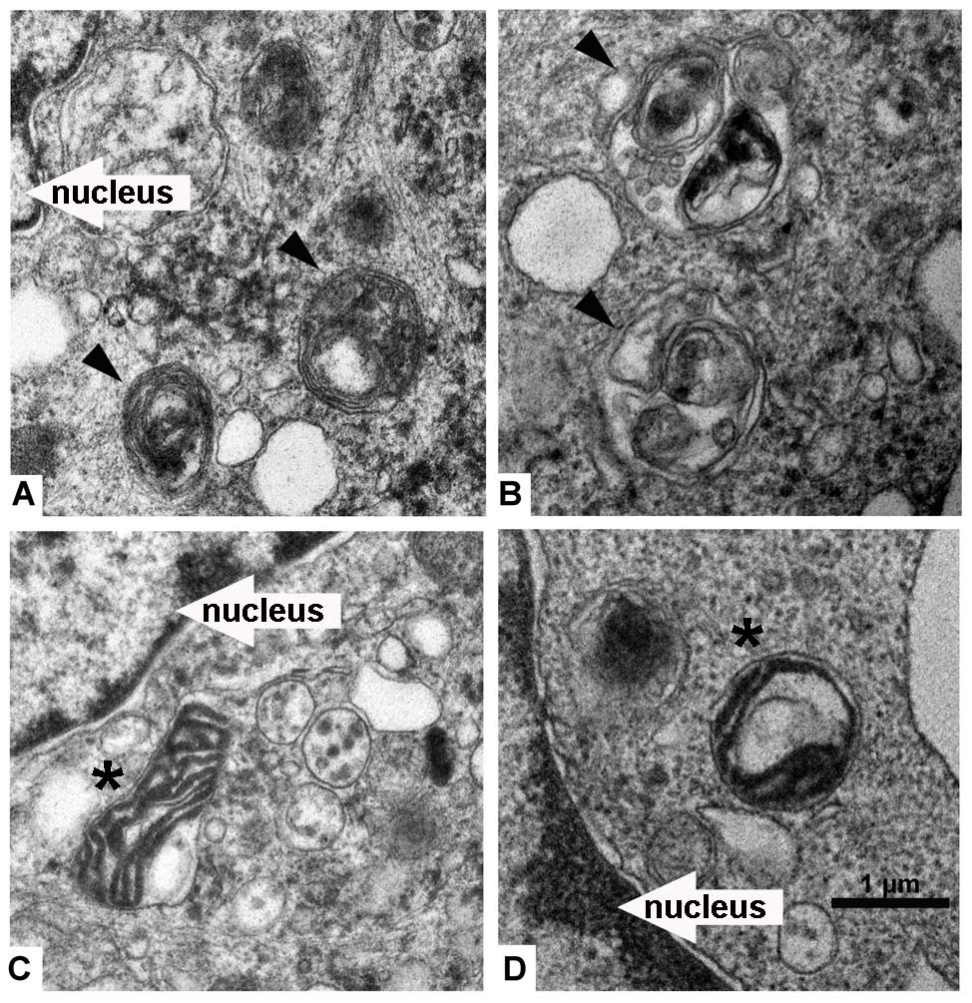
Morphology of autophagosomes in EF25-(GSH)_2_-treated HepG2 cells. HepG2 cells were treated with 20 µmol/L EF25-(GSH)_2_ for 16 h and observed under transmission electron microscopy. (A) and (B), multimembranous autophagic vacuoles engulfing cytoplasmic components are indicated with black arrowheads. (C) and (D), autophagic vacuoles containing a mitochondrion are indicated with black asterisk.

The ultrastructural details of HepG2 cells treated with 20 µmol/L EF25-(GSH)_2_ for 16 hours were further examined by transmission electron microscopy. Typical multimembrane autophagic vesicles engulfing cytoplasmic components and organelles were identified in the cytoplasm (Fig. 4).

**Figure 5:**
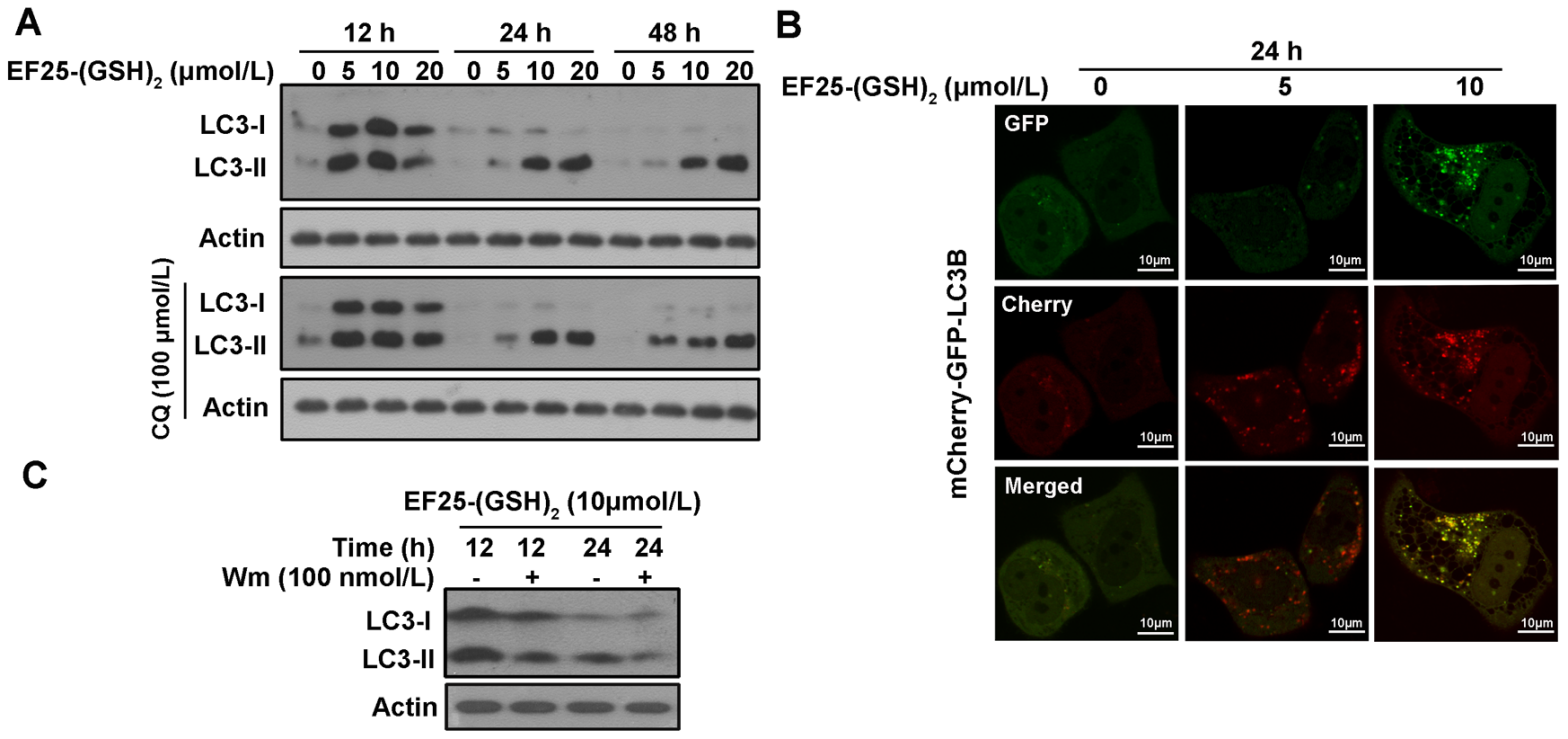
EF25-(GSH)_2_ induced autophagy in HepG2 cells. (A) Western blot analysis of the LC3B expression in HepG2 cells treated with EF25-(GSH)_2_ at varying concentrations for 12 to 48 h with or without chloroquine (CQ, 100 µmol/L). (B) The cellular distribution of mCherry-GFP-LC3B in HepG2 cells treated with EF25-(GSH)_2_ at different concentrations for 24 h was examined under a laser confocal microscope. (C) Lysates from HepG2 cells incubated with 10 µmol/L EF25-(GSH)_2_ for 12 or 24 h pretreated with or without wortmannin (Wm, 100 nmol/L, pretreated for 2 h) were analyzed by Western blotting for LC3B expression level.

To further confirm whether EF25-(GSH)_2_ triggered autophagy in HepG2 cells, we examined the expression of the two forms of microtubule-associated protein 1 light chain 3 (LC3). In the process of autophagy, LC3-I residing in the cytosol is modified to LC3-II, which binds to the autophagosome membrane. Thus, the degree of LC3-I to LC3-II conversion correlates to the extent of autophagosome formation [25]. EF25-(GSH)_2_ treatment obviously increased the expression level of both LC3-I and LC3-II as early as 12 hours post-treatment, but the bands corresponding to LC3-I were weakened and there was no obvious augmentation in the LC3-II expression when EF25-(GSH)_2_ treatment was prolonged or the dosage was increased, indicating that the lack of conversion of LC3-I to LC3-II may due to incomplete autophagy (Fig. 5A).

The increase in LC3-II expression can be associated with either an enhanced formation of autophagosome or an impaired autophagic degradation [26]. Chloroquine (CQ) is a lysosomal trophic agent that raises the lysosomal pH and, hence, blocks autophagy at the late stages [27]. Accordingly, CQ was used to test if EF25-(GSH)_2_ can induce complete autophagic flux [28], [29]. In cells treated with 5 µmol/L EF25-(GSH)_2_, the LC3-II showed progressive accumulation in the presence of CQ at 24 h and 48 h. However, at 10 and 20 µmol/L, EF25-(GSH)_2_-treated samples with and without CQ were indistinguishable with respect to LC3-II expression (Fig. 5A). This data indicates that autophagy flux was achieved at 5 µmol/L EF25-(GSH)_2_ but was blocked at 10 and 20 µmol/L.

In addition, we examined the localization of autophagosome-specific protein LC3B in HepG2 cells treated with EF25-(GSH)_2_ for 24 hours using Cherry-GFP-LC3B plasmid. When autophagy is induced, exogenous LC3 distributes to the membrane of autophagosomes and shows characteristic green (GFP) or red (mCherry) dots. Because GFP is acid-labile, only mCherry red fluorescence can be seen in autophagolysosmes, while the neutral structures display both green and red fluorescence [30]. In untreated cells, mCherry-GFP-LC3B showed a homogeneous distribution, whereas the EF25-(GSH)_2_-treated cells showed fluorescent dots. At 5 µmol/L, the cells exhibit mostly only red dots, suggestive of autophagic degradation. Meanwhile, at 10 µmol/L, cells expressed double-tagged fusion proteins indicating that autophagic degradation was blocked (Fig. 5B). The data coincide well with the immunoblot analysis of LC3B in the presence of CQ.

### 5. EF25-(GSH)_2_ induced G_2_/M cell cycle arrest and apoptosis in HepG2 cells

**Figure 6:**
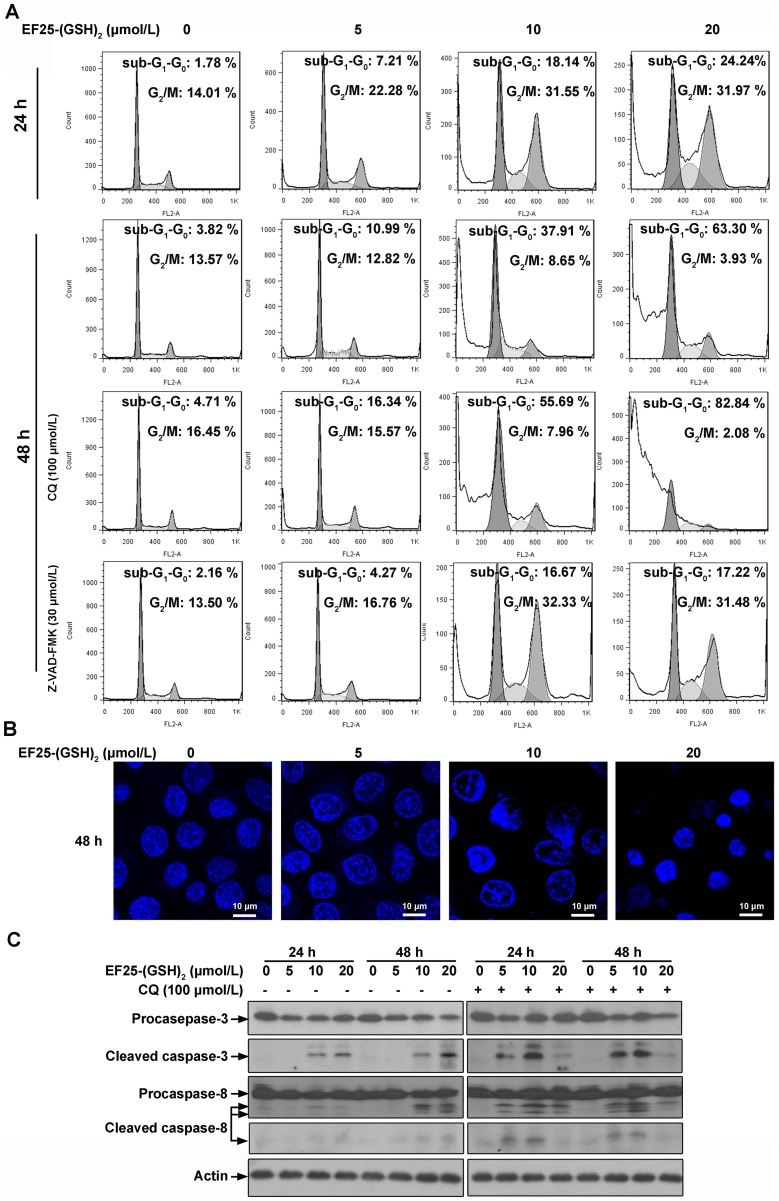
The apoptosis in HepG2 cells triggered by EF25-(GSH)_2_ in the presence or absence of CQ/Z-VAD-FMK. (A) HepG2 cells were treated with various concentrations of EF25-(GSH)_2_ for 24 h and 48 h with or without chloroquine (CQ, 100 µmol/L)/Z-VAD-FMK (30 µmol/L, pretreated for 2 h) and then analyzed for DNA content (propidium iodide, PI) and cell cycle distribution. Apoptosis was measured as the percentage of cells containing hupodiploid quantities of DNA (sub-G_1_-G_0_ peak). Percentage of cells within the sub-G_1_-G_0_ and G_2_/M stages is shown for each data point. Graphs are representative of data collected from three independent experiments. (B) HepG2 cells incubated with increasing concentrations of EF25-(GSH)_2_ for 48 h were stained with 4, 6-diamidino-2-phenylindole (DAPI) and examined by laser confocal microscopy. Untreated HepG2 cells showed uniformly stained nuclei, while EF25-(GSH)_2_-treated cells exhibited chromatin condensation in a concentration-dependent manner. (C) Lysates from HepG2 cells incubated with increasing concentrations of EF25-(GSH)_2_ for 24 or 48 h with or without chloroquine (CQ, 100 µmol/L) were analyzed by Western blotting for both full length and cleaved caspase-3 and caspase-8 expression levels.

Curcumin and its analogs have consistently been reported to induce apoptosis [31], [32]. To determine whether EF25-(GSH)_2_ acts similarly in HepG2 cells, the DNA content of permeabilized PI-stained cells was examined by flow cytometry at 24 h and 48 h post-treatment. The cell cycle analysis showed obvious G_2_/M cell cycle arrest at 24 h, and the percentage of cells in sub-G_1_-G_0_ was greatly augmented at 48 h in a concentration-dependent manner (Fig. 6A).

DAPI staining of the nuclei also indicated that EF25-(GSH)_2_-treated cells underwent apoptosis, the extent of which was concentration dependent. Untreated HepG2 cells showed uniformly stained nuclei, while nuclei of EF25-(GSH)_2_-treated cells were condensed or fragmented with strengthened fluorescence (Fig. 6B).

These findings were further confirmed by analysis of the expression level of cleaved caspase-8 and caspase-3, both of which were augmented at 24 h post-treatment and maintained a high level up to 48 h at concentrations of 10 µmol/L and 20 µmol/L, whereas caspase activation was undetectable at 5 µmol/L (Fig. 6C).

### 6. Wortmannin advanced EF25-(GSH)_2_ induced cell death in HepG2 cells in the early period

**Figure 7:**
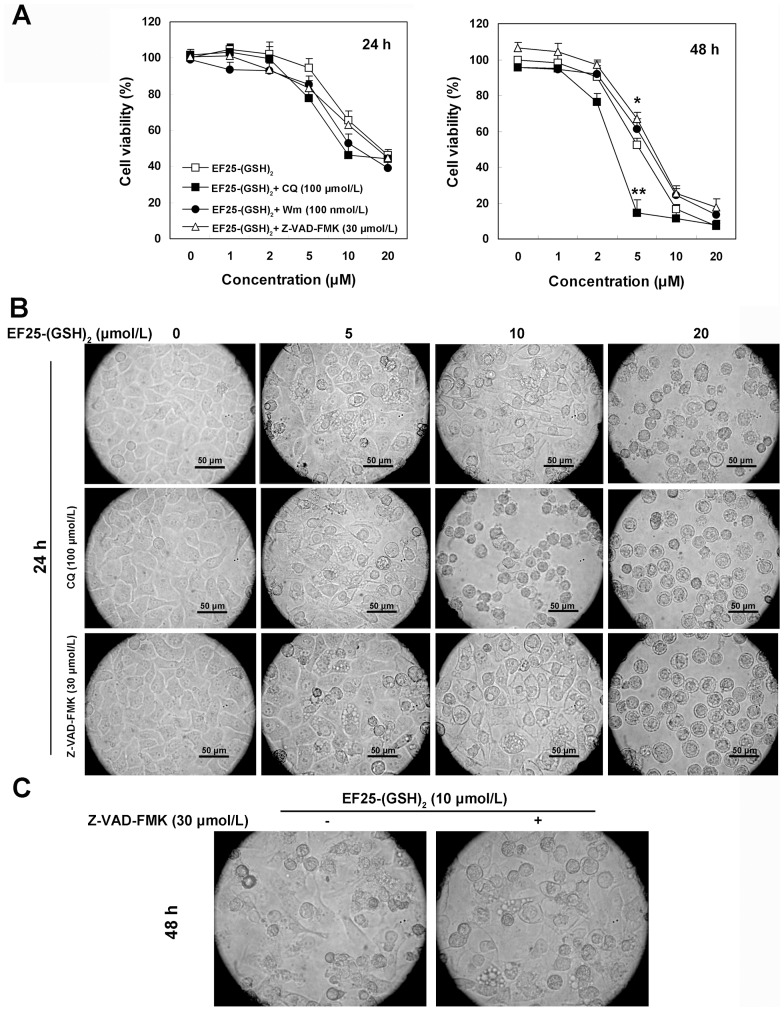
The effect of Wm, CQ and Z-VAD-FMK on the cytotoxicity and morphological changes induced by EF25-(GSH)_2_ in HepG2 cells. (A) Cell viability was determined by the MTT assay after treatment with increasing concentrations of EF25-(GSH)_2_ for 24 h or 48 h in the absence or presence of CQ (100 µmol/L)/Wm (100 nmol/L, pretreated for 2 h)/Z-VAD-FMK (30 µmol/L, pretreated for 2 h). *, p<0.001, EF25-(GSH)_2_ plus Z-VAD-FMK vs. EF25-(GSH)_2_ alone. **, p<0.001, EF25-(GSH)_2_ plus CQ vs. EF25-(GSH)_2_ alone. (B) Representative light microscopic images of HepG2 cells treated with various concentrations of EF25-(GSH)_2_ for 24 h in the absence or presence of CQ (100 µmol/L)/Z-VAD-FMK (30 µmol/L, pretreated for 2 h). (C) Representative light microscopic images of HepG2 cells treated with 10 µmol/L EF25-(GSH)_2_ for 48 h in the absence or presence of Z-VAD-FMK (30 µmol/L, pretreated for 2 h).

Autophagy modulation is a double edged sword in cancer treatment, possibly due to various cellular settings [33]. To test whether autophagy contributed to or hampered EF25-(GSH)_2_ promoted HepG2 cell death, an inhibitor of autophagic sequestration (wortmannin (Wm)) was used to block autophagy at the early stages [26]. In the presence of 100 nmol/L Wm, the expression levels of both LC3B I and II types were largely reduced, indicating that Wm was effective in inhibiting EF25-(GSH)_2_-induced autophagy formation (Fig. 5C). Wm at 100 nmol/L was only slightly toxic to HepG2 cells but clearly promoted the EF25-(GSH)_2_-indued death process in the first 24 hours as evidenced by earlier cell shrinkage, rounding up (data not shown) and a 7–12% fall in cell viability examined by the MTT assay. However, as time progressed, the MTT assay at 48 h showed a slight increase rather than a further decrease of cell viability in Wm-pretreated cells. This indicates that Wm treatment advanced cell death only in the early period but had no obvious effect on the ultimate cytotoxicity of EF25-(GSH)_2_ (Fig. 7A).

### 7. Knockdown of Atg5 and Beclin-1 expression did not rescue EF25-(GSH)_2_-treated HepG2 cells

**Figure 8:**
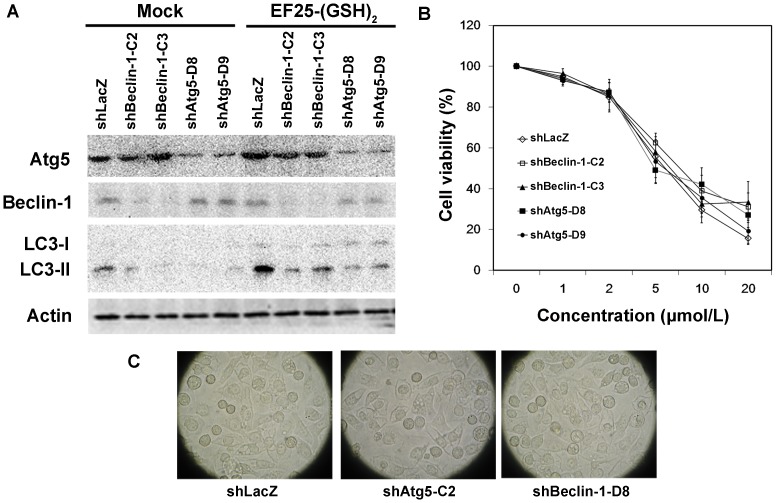
Knockdown of Atg5 and Beclin-1 expression does not rescue EF25-(GSH)_2_-treated HepG2 cells. (A) HepG2 cells respectively transduced with shLacZ-, shBeclin-1-C2-, shBeclin-1-C3-, shAtg5-D8- and shAtg5-D9-lentivirus were mock-, or treated with 10 µmol/L EF25-(GSH)_2_ for 24 h. Cells lysates were analyzed by Western blotting with antibodies against Atg5, Beclin-1, LC3 or actin, as indicated. (B) For HepG2 cells respectively transduced with shLacZ-, shBeclin-1-C2-, shBeclin-1-C3-, shAtg5-D8- and shAtg5-D9-lentivirus, cell viability was determined by MTT assay after treatment with increasing concentrations of EF25-(GSH)_2_ for 48 h. (C) HepG2 cells respectively transduced with shLacZ-, shBeclin-1-C2- and shAtg5-D8-lentivirus were treated with 10 µmol/L EF25-(GSH)_2_ for 24 h and observed under the light microscope.

In order to avoid the non-specific effect of Wm, we knocked down the cellular expression of two autophagy essential genes, Atg5 and Beclin-1, separately, using specific small hairpin RNAs (shRNA) delivered by the lentiviral expression system. The cells were transduced with lentivirus expressing the shRNA targeting LacZ, Atg5 or Becllin 1, and were selected with puromycin. Puromycin-selected cells were then treated with 10 µmol/L EF25-(GSH)_2_ for 24 h. Atg5- and Beclin-1-knockdown was evident by reduced expression level of Atg5 and Beclin-1, respectively. Furthermore, both Atg5- and Beclin-1-knockdown resulted in the attenuated expression level of LC3II visualized with immunoblotting (Fig. 8A). The MTT assay showed no obvious distinction in cell viability between LacZ-knockdown and Atg5/Beclin-1-knockdown HepG2 cells, which produced similar results with Wm (Fig. 8B). Furthermore, Atg5/Beclin-1-knockdown did not prevent the extensive cytoplasmic vacuolization induced by EF25-(GSH)_2_, suggesting that this phenomenon is not directly induced by autophagic degradation (Fig. 8C).

### 8. CQ promoted cytoplasmic vacuolization, apoptosis and cell death induced by EF25-(GSH)_2_ in HepG2 cells

It has been previously reported that inhibition of autophagy at different stages of the process can lead to distinct results [34], [35]. In our study, inhibition of autophagy at an early stage by Wm did not significantly alter either the extent of cytoplasmic vacuolization or the final cell viability at 48 h. However, the late stage inhibitor CQ not only advanced the cell death process but also significantly enhanced cytoplasmic vacuolization, apoptosis and cytotoxicity induced by EF25-(GSH)_2_. This combination effect of CQ was most dramatic by treatment of EF25-(GSH)_2_ at 5 µmol/L.

When exposed to 5 µmol/L EF25-(GSH)_2_ alone, only a small portion of cells was moderately vacuolated, but in the presence of CQ the majority of cells underwent extensive vacuolization to an extent similar to that caused by 10 µmol/L EF25-(GSH)_2_ (Fig. 7B). This observation indicates that EF25-(GSH)_2_-induced autophagy exhibits a cytoprotective role at lower concentration.

The MTT assay showed that CQ enhanced the effectiveness of EF25-(GSH)_2_ within a 24-h period, continued to 48 h and proved especially clear-cut at the concentration of 5 µmol/L where cell viability dramatically dropped from 52.4% to 14.5% after 48 h treatment (Fig. 7A).

We also found that combining CQ with EF25-(GSH)_2_ greatly augmented the apoptosis rate evidenced by a large increase in the percentage of cells in the sub-G_1_-G_0_ stage in a concentration-dependent manner (Fig. 6A).

In the presence of CQ, the expression level of activated caspase-3 increased at concentrations of 5 µmol/L and 10 µmol/L, but unexpectedly decreased at 20 µmol/L. Similarly, the expression level of cleaved caspase-8 was clearly increased by CQ treatment except at a concentration of 20 µmol/L EF25-(GSH)_2_ at 48 h (Fig. 6C).

### 9. Z-VAD-FMK prolonged vacuolization and G2/M cell cycle arrest partially rescues HepG2 cells from EF25-(GSH)2 toxicity

To determine whether caspase activation plays a crucial role in EF25-(GSH)_2_-induced cytotoxicity, the pan caspase inhibitor Z-VAD-FMK was employed. In the presence of this compound at 48 h post-treatment, cell cycle analysis showed a clear decrease of cells in the sub-G_1_-G_0_ stage especially at concentrations of 10 (from 37.9% to 16.7%) and 20 µmol/L (from 63.3% to 17.2%) (Fig. 6A). These data indicate that apoptosis induced by EF25-(GSH)_2_ is primarily caspase- and concentration-dependent and partially caspase-independent to the extent of about 17%.

However, compared to the 21.2% (10 µmol/L) and 46.1% (20 µmol/L) decrease of apoptotic cells in the presence of Z-VAD-FMK at 48 h, only a 6.3% (10 µmol/L) and 19.3% (20 µmol/L) rise in cell viability was observed when pretreated with Z-VAD-FMK as examined by the MTT assay at 48 h (Fig. 7A), indicating the operation of non-apoptotic cell death. The latter was accompanied by prolonged cytoplasmic vacuolization and G_2_/M cell cycle arrest.

The extent of cytoplasmic vacuolization was not significantly enhanced in the presence of Z-VAD-FMK (Fig. 7B), but the period of vacuolization was prolonged beyond the 48 h treatment. Cells exposed to 10 µmol/L EF25-(GSH)_2_ alone avoided vacuolization and begin to shrink at 48 h. In contrast, cells exposed to co-treatment of 10 µmol/L EF25-(GSH)_2_ and 30 µmol/L Z-VAD-FMK exhibited extensive vacuolization (Fig. 7C).

Cell cycle analysis showed obvious G_2_/M cell cycle arrest in the presence of Z-VAD-FMK at 48 h post-treatment, which is similar to what was observed at 24 h post-treatment with EF25-(GSH)_2_ alone, indicating that Z-VAD-FMK prolonged the status of G_2_/M cell cycle arrest induced by the EF25 conjugate (Fig. 6A).

## Discussion

Effective and less toxic alternative chemotherapeutic agents against HCC are needed to address the emerging problem of drug resistance and severe side effects [2]. Widely used drugs like cisplatin exhibit no selectivity for malignant cells, while natural compounds like curcumin, which possess a good safety profile, exert inadequate effectiveness [7].

By contrast, our *in vitro* and *in vivo* data show the novel compound EF25-(GSH)_2_ to exert preferential toxicity toward HCC cells, offering potential as a promising anti-HCC therapeutic agent. In addition, the double GSH conjugation successfully solves the problems of instability and water insolubility which limits the usage of curcumin and its analogues [8].

Basic and clinical studies have clearly established the importance of apoptosis in therapeutic tumor-cell death, but many notable studies have confirmed that other forms of cell death are crucial for effective cancer therapy, and that apoptosis is not the comprehensive answer especially when dealing with the whole tumor instead of isolated tumor cells [36]. We found that the action of EF25-(GSH)_2_ is complex in terms of which death pathways are involved. In EF25-(GSH)_2_ treated HepG2 cells, autophagy and apoptosis were detected and extensive cytoplasmic vacuolization was observed. These events do not occur independently, but are closely connected.

The role of autophagy in cancer therapy is complex and depends on the specific cellular setting and treatment scenario. Under some circumstances, autophagy rescues cells under stress conditions and, in this sense, may suppress apoptosis and/or other types of cell death. In other scenarios, irreversible self-destruction caused by massive autophagy leads to cell demise [33]. To investigate the exact role of autophagy in chemotherapy, autophagy inhibitors at different stages have been previously employed. Interestingly, the blockade of autophagy at an early or late stage has been reported by some groups to cause different effects. For example, the late stage inhibition by Bafilomycin A1 was found to enhance apoptosis and cell death, whereas inhibition of autophagy at early stages using 3-MA failed to do so [34], [35].

Our autophagy inhibitor data using Wm and CQ also show different effects. Inhibition of autophagy at early stages by Wm advanced the cell death process during early phases of EF25-(GSH)_2_ treatment, but altered the final toxicity insignificantly. In contrast, CQ greatly enhanced cytoplasmic vacuolization, apoptosis and cell death. These data suggest that autophagy does not directly execute cell death through extensive digestion of cellular cytoplasm, but exhibits a cytoprotective role and functions as a fail-safe response to the stressful condition induced by EF25-(GSH)_2_. In spite of this, protective autophagic degradation is only operative at low concentrations and is blocked by the action of the compound itself at higher and more cytotoxic concentrations.

However, we found that blocked autophagy contributes to cell death induced by EF25-(GSH)_2_. In EF25-(GSH)_2_-treated HepG2 cells, autophagy degradation blockage is accompanied by extensive cytoplasmic vacuolization. The latter phenomenon was found in tumor cells under various chemotherapeutic treatments. Although the cells present with a common morphology, various mechanisms were proposed [14], [37]–[39]. Hence, we conclude that accumulation of autophagosomes instead of autophagic degradation promotes the formation of extensive cytoplasmic vacuolization and subsequent cell death. In some effective cancer therapies, impaired autophagy has been observed [40], which may cause metabolic dysfunction and make cells more susceptible to other types of cell death. Notably, preclinical investigations combining the autophagy late stage inhibitor hydroxychloroquine (HCQ) with various chemotherapies has already entered clinical trials [41].

With EF25-(GSH)_2_ alone, the number of cells within the G_2_/M stage was found to be augmented at a 24 h post-treatment and then largely diminished at 48 h when the number of apoptotic cells greatly soared. Non-apoptotic cell death occurs when the process from G_2_/M stage arrest to caspase-dependent apoptosis is blocked by Z-VAD-FMK, implying that cells arrested at G_2_/M have already reached a “point of no return” in the lethal process and that caspase activation may not be necessary. Notably, this result suggests that for cells failed to undergo apoptosis, EF25-(GSH)_2_ induced cell death through non-apoptotic mechanisms, although less effectively without the participation of caspase activation.

As expected, activation of both caspase-3 and caspase-8 was enhanced under co-treatment of CQ and 5 µmol/L or 10 µmol/L EF25-(GSH)_2_. However, caspase activation was undetectable with co-treatment of CQ and 20 µmol/L EF25-(GSH)_2_, whereupon the apoptosis rate soared, indicating that the apoptosis in this scenario is mainly caspase-independent. Cell cycle analysis in the presence of the pan-caspase inhibitor Z-VAD-FMK suggests that EF25-(GSH)_2_ alone causes mainly caspase-dependent apoptosis, but also partially caspase-independent apoptosis.

**Figure 9:**
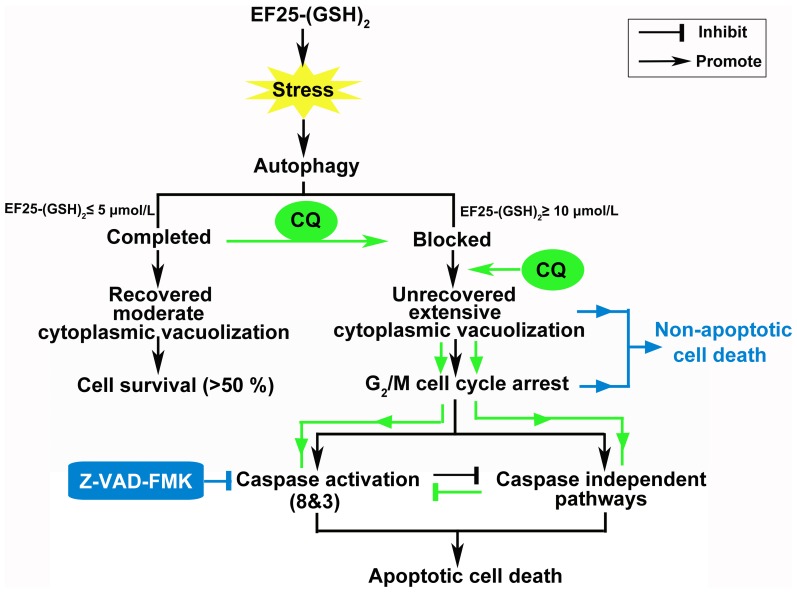
Working model of the mechanisms of EF25-(GSH)_2_-induced cell death in HepG2 cells. Stress induced by EF25-(GSH)_2_ promotes autophagy in HepG2 cells. When treated with EF25-(GSH)_2_ at concentrations of 5 µmol/L or lower, cells experienced full-scale autophagy that displayed moderate cytoplasmic vacuolization, ultimate recovery and partial rescue of cells from the resulting stress. In contrast, the protective autophagy was blocked in cells treated with EF25-(GSH)_2_ at concentrations of 10 µmol/L or higher which led to massive cytoplasmic vacuolization. The latter cells arrested in the G_2_/M phase succumbed to both caspase-dependent and caspase-independent cell death. EF25-(GSH)_2_ treatment alone led mainly to caspase-dependent apoptotic cell death, but also to a significant proportion of caspase-independent apoptosis. The action of EF25-(GSH)_2_ could be modulated by CQ (green) and Z-VAD-FMK (blue). Co-treatment of EF25-(GSH)_2_ with CQ promoted autophagy blockage and cytoplasmic vacuolization, which then enhanced apoptosis for both caspase-dependent and caspase-independent mechanisms. Co-treatment of EF25-(GSH)_2_ with Z-VAD-FMK inhibited caspase activation and subsequently blocked the caspase-dependent apoptotic death route. Thus, cells were trapped by cytoplasmic vacuolization and G_2_/M cell cycle arrest, which eventually led to non-apoptotic cell death.

To sum up, we propose an anti-hepatoma mechanistic model for EF25-(GSH)_2_ in Figure 9. When treated with EF25-(GSH)_2_ at a concentration of no more than 5 µmol/L, cells experience successful autophagic degradation. In this case, moderate cytoplasmic vacuolization takes place followed by subsequent recovery, which partially rescues cells from a stressed condition. However, EF25-(GSH)_2_ at a concentration of 10 µmol/L or higher leads to impaired autophagy during which the autophagic degradation step is blocked and followed by massive cytoplasmic vacuolization. At this point, cells undergo both caspase-dependent and caspase-independent apoptosis. EF25-(GSH)_2_ treatment alone leads mainly to caspase-dependent apoptosis accompanied by partial caspase-independent apoptosis. Co-treatment with CQ stimulates autophagosome accumulation and cytoplasmic vacuolization, which then promotes both caspase-dependent and caspase-independent apoptosis. Z-VAD-FMK inhibits caspase activation and subsequently blocks the path to apoptotic death. In this case, the status of vacuolization and G_2_/M cell cycle arrest is prolonged and eventually leads to non-apoptotic cell death.

In conclusion, our results show that the novel curcumin analog EF25-(GSH)_2_ has promising potential as a low toxicity chemotherapeutic agent for HCC. Similar to curcumin, the anti-tumor action of EF25-(GSH)_2_ involved in autophagic, apoptotic and non-apoptotic mechanisms would broaden its application. The combination of EF25-(GSH)_2_ with chloroquine is suggested to provide a safer and more effective treatment for HCC.

## Supporting Information

Figure S1 **MS/HR-ESI-MS spectra of EF25-(GSH)_2_.**
(TIF)

Figure S2 **Overlay of EF25 (blue) and EF25-(GSH)_2_ (green) 1H NMR spectra in DMSO-d6. EF25 1H NMR spectrum in DMSO-d6: solvent peak at 2.5 ppm (light yellow); 10.2(s) (OH), 7.9 ( = C–H), 6.8–7.3 (aromatic) ppm.**
(TIF)

Figure S3 **Overlay of EF25 in DMSO-d6 (blue) and EF25-(GSH)_2_ in D20 (green), buffer pH7, 1H NMR spectra.**
(TIF)
