# Supplementary figures and images for: Autophagy and Apoptosis in Hepatocellular Carcinoma Induced by EF25-(GSH)2: A Novel Curcumin Analog
Source: PLoS One. 2014 Sep 30;9(9):e107876. doi: 10.1371/journal.pone.0107876 (PMC4182433; doi:10.1371/journal.pone.0107876)

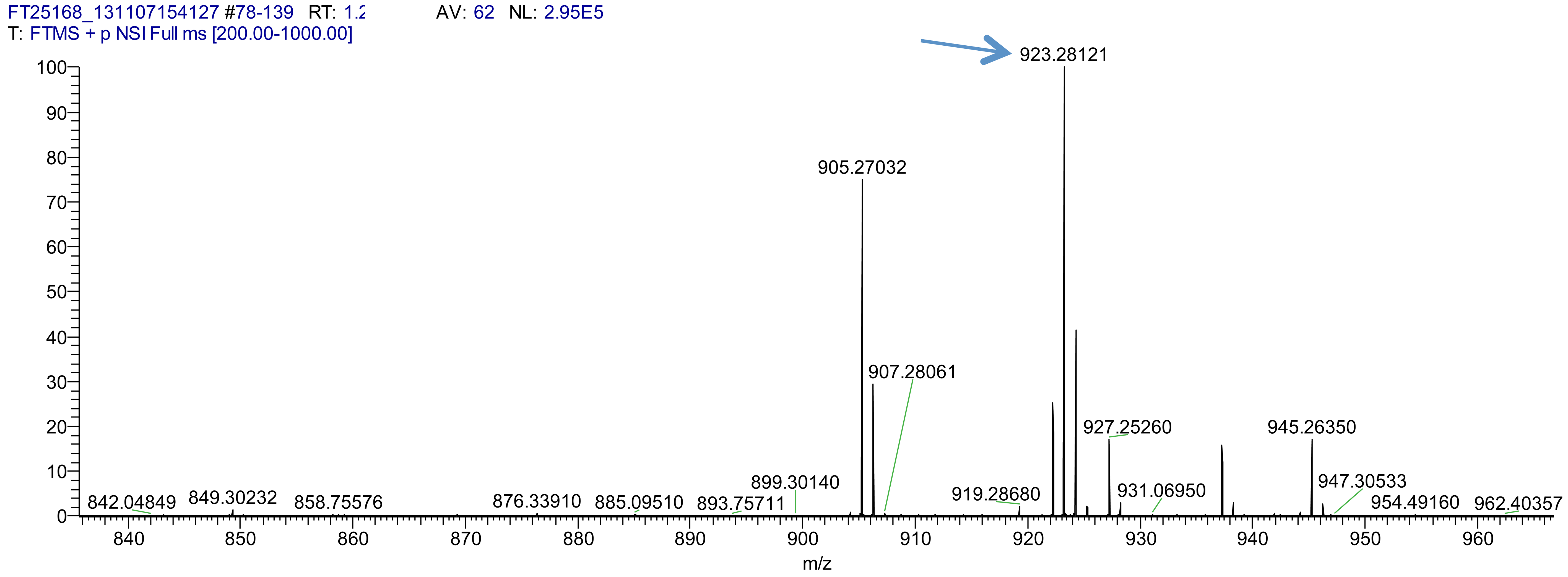

Supplement: Figure S1 — MS/HR-ESI-MS spectra of EF25-(GSH)2. (TIF) [file pone.0107876.s001.tif]

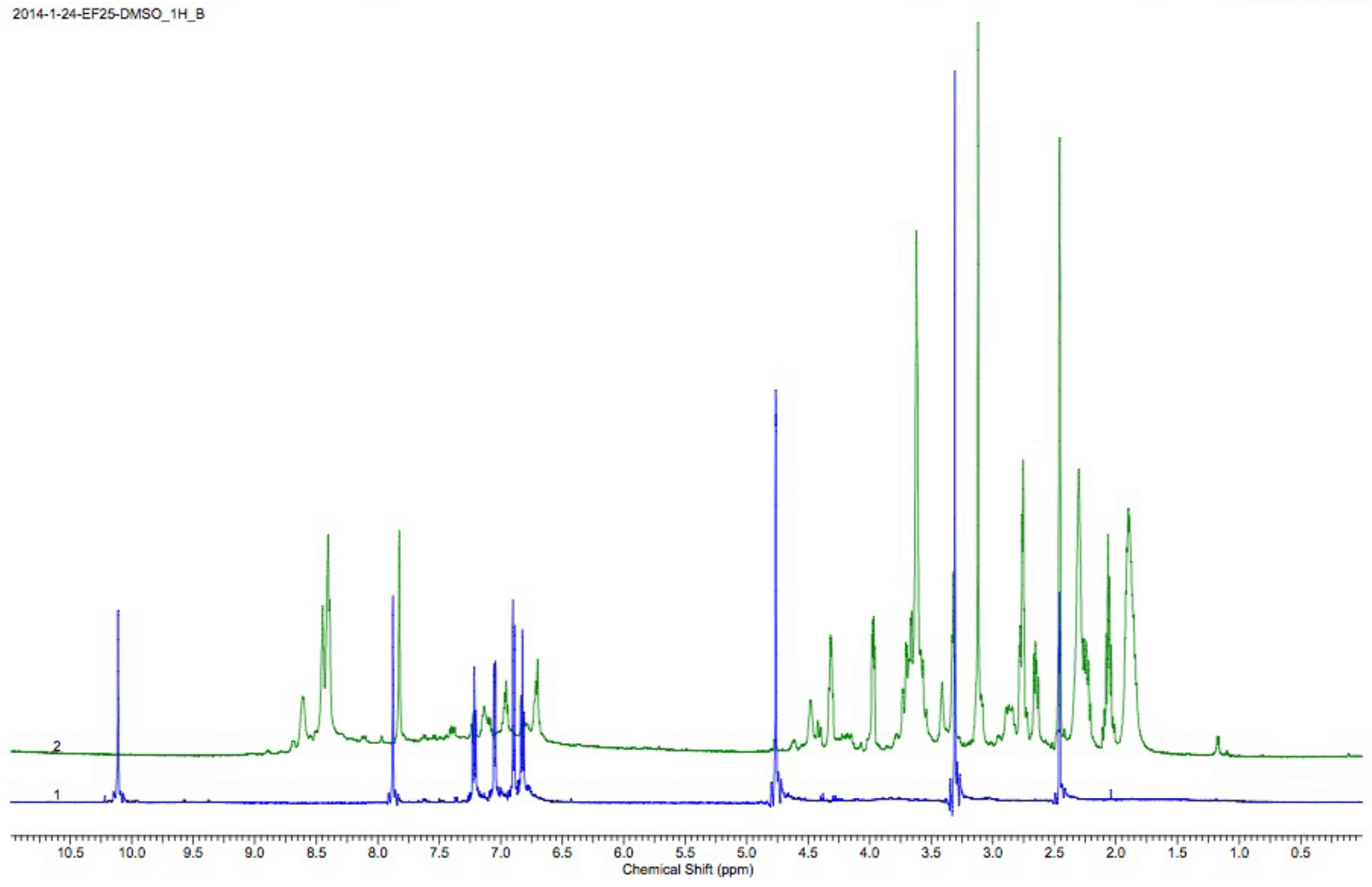

Supplement: Figure S2 — Overlay of EF25 (blue) and EF25-(GSH)2 (green) 1H NMR spectra in DMSO-d6. EF25 1H NMR spectrum in DMSO-d6: solvent peak at 2.5 ppm (light yellow); 10.2(s) (OH), 7.9 ( = C–H), 6.8–7.3 (aromatic) ppm. (TIF) [file pone.0107876.s002.tif]

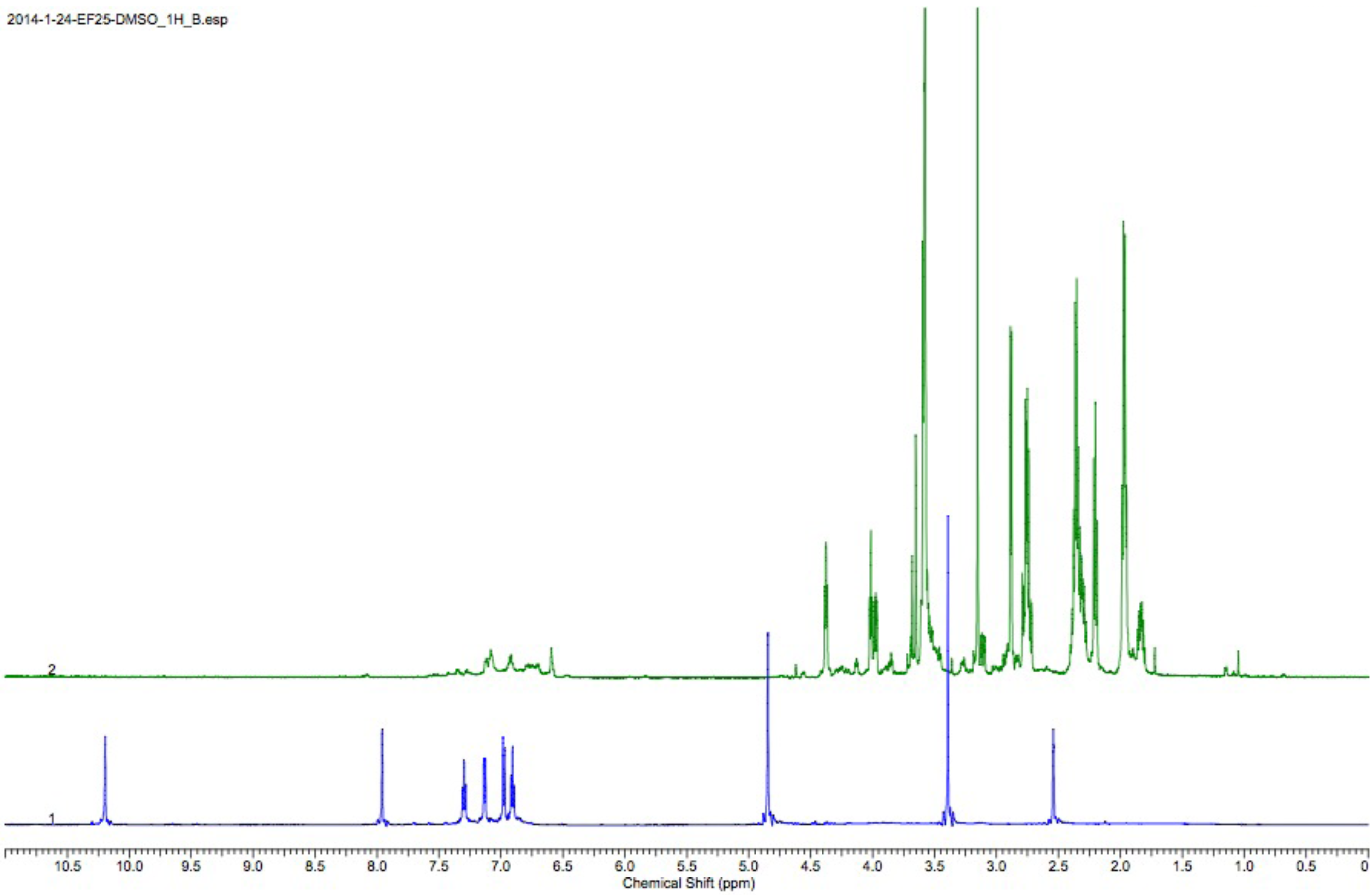

Supplement: Figure S3 — Overlay of EF25 in DMSO-d6 (blue) and EF25-(GSH)2 in D20 (green), buffer pH7, 1H NMR spectra. (TIF) [file pone.0107876.s003.tif]
